# Factors that influence the clinical utilization of the nursing process at a hospital in Accra, Ghana

**DOI:** 10.1186/s12912-017-0228-0

**Published:** 2017-06-09

**Authors:** Joana Agyeman-Yeboah, Kwadwo Ameyaw Korsah, Jane Okrah

**Affiliations:** 1 37 Military Hospital, Accra, Ghana; 2 University of Ghana, P.O. Box LG43, Legon, Ghana; 3 UNAIDS, Accra, Ghana

**Keywords:** Nursing-care plan, Nursing process, Clinical utilization

## Abstract

**Background:**

The nursing process is a tool that is recommended for use by all professional nurses working in Ghana, in order to provide nursing care. However, there is currently a limited use of this tool by nurses in Ghana. The purpose of this research study was to explore the various factors that influence the utilization of this nursing process.

**Method:**

An exploratory descriptive qualitative-research design was employed. Ten participants were involved by using the purposive sampling method. A semi-structured interview guide was used to collect the data from the research participants; and the data were analysed by using content analysis. One main theme, with five subthemes, emerged from the analysis.

**Results:**

It was found that there are factors, such as nurses not having a better understanding of the nursing process, whilst in school; the absence of the care plan in the ward, as well as the lack of adequate staff, with limited time being available for coping with contributed to the non-usage of the nursing process.

**Conclusions:**

We conclude that the clinical utilization of the Nursing process at the clinical setting is influenced by lack of understanding of Nurses on the Nursing process and care plan as well as lack of adequate nurses and time. We recommend that the care-plan form be made officially a part of the admission documents. Furthermore, the nursing administration should put measures in place to provide nurses with the needed resources to implement the nursing process. Additionally, they should ensure that the care-plan forms and other resources needed by the nurses are regularly and adequately provided. Nurses should further see the nursing process as a means of providing comprehensive care to their patients and addressing their specific problems. They should therefore make time despite their busy schedules to use it in order to improve quality of care and the image of nursing in Ghana.

**Electronic supplementary material:**

The online version of this article (doi:10.1186/s12912-017-0228-0) contains supplementary material, which is available to authorized users.

## Background

The nursing process has been defined as the series of critical thinking activities that are used by nurses as they care for their patients [1]. These activities define a nursing model of care, differentiating nursing from other helping professions [1]. The nursing process consists of interconnected steps; and it is an organized and self-motivated way of giving nursing care to patients. It encourages humanistic, outcome-focused, cost-effective care; and it is based on the belief that, as we plan and convey care, we must also consider the exceptional values, concerns, and desires of the consumer, who may be an individual, a family, or a community [2].

The utilization of the nursing process and a nursing diagnosis has been identified as critical to nursing practice [3]. Some of the factors affecting the use of the nursing process have been studied among nurses working in various health institutions. Several factors were found to interfere with the efficient implementation of the nursing process. Operational difficulties involved in the systematization of nursing care in practice, such as, a lack of knowledge of the steps involved in the process, an excessive number of tasks assigned to the nursing team, the poor quality of professional education, and inadequate reports on the physical examination related to the disease, are among these factors [4].

The nursing process and the resulting written plan of care improve communication through the sharing of knowledge and information between departments and shifts, as well as ensuring better communication with the multidisciplinary team [5].

Despite the benefits of using the nursing process and a written care plan, there is still a limited use of these tools by nurses in Ghana. Since the introduction of the nursing process in Ghana in 1970, there has been a decrease in the use of the care plan among professional nurses at the clinical area. Currently, there is no evidence of the use of the nursing process; since the care plan form is not common among the admission documents used at the clinical area in Ghana (Addison & Commey: Use of the nursing process among professional nurses, unpublished). The question that one may well ask is: What are the various factors that are preventing the nurses from utilizing the nursing process in the management of their patients? The purpose of this study was thus to explore those factors that influence the clinical utilization of the nursing process.

## Methods

An exploratory, descriptive qualitative design was employed; and the study was carried out at a hospital in Accra. The research population comprised all the professional nurses, who were registered by the Nursing and Midwifery council (NMC) of Ghana. Nurses, who had at least 1 year of working experience; and who were full-time employees of the 37 Military Hospital, were included. The participants consisted of three staff nurses, two senior staff nurses and five nursing officers. Their ages ranged from 27 years to 41 years; and the number of years that the participants had been practising also ranged from 4 years to 9 years.

The researchers sought permission from the in-charges of the various wards – after the essence of the research was explained to them – as well as to the nurses. The researchers also approached potential participants who fell within the inclusion criteria; and they invited them to be part of the research. Those who expressed interest in the study were briefed on the purpose of the study, including the process of the data collection. They were informed that the data would be recorded on a voice recorder; and they were assured of their confidentiality, as well as proper management of the data. The permission and consent of each participant was sought when using the voice recorder. They were assured of their rights and the freedom to withdraw from the study at any time; if they did not wish to continue. A consent form was given to those who were willing to participate in the study for them to read; and further explanations were given about the form. Two consent forms were signed by each participant; and a copy of the original signed form was given to them to keep.

An appropriate date, time and place for the interview was arranged with the participants; and this was based on the preference of the participants. The data were collected via a semi-structured interview guide (attached as an Additional file 1), which was first piloted at a different hospital, in order to ensure the credibility of the data. Each interview lasted for approximately 45 min – 1 h. Thus, sufficient time was spent with the participants for maintaining rapport and building a trusting relationship with them to ensure that credible data were obtained.

After interviewing the tenth participant, no new information was forthcoming; thus, it was assumed that the data had reached saturation.

The data were analysed by using the latent-content analysis method [6]; and it was conducted simultaneously with the interviews. Verbatim transcriptions were obtained after an interview session. The data were then coded where persistent words, phrases or concepts within the data were identified. Comparisons among the data were found. After the coding, the data were collated with similar codes; and they were developed into categories.

A summary of each category was written down; and one main theme was developed after a careful analysis of the categories was carried out.

## Results

From the data analysis, the main theme that emerged was those factors that impede the ability of nurses to use the nursing process. Five sub-themes emerged; and these are described below.

### The nursing process was not taught well at nurses’ training schools

According to the participants, one factor that influenced the ability of the nurses to use the nursing process after qualifying as a professional nurse, is how he or she were taught the nursing process at school. Some of the participants alleged that some of the teachers who taught the nursing process at the Nurses Training Colleges (NTC) and at the School of Nursing at the Universities did not understand the nursing process very well; hence, they were not able to teach it well. Consequently, the students do not readily acquire an understanding of the general concept of the nursing process; and do not acquire the needed skills to enable them to put the knowledge they possess into practice after the completion of their schooling.

This was expressed in various forms by the participants. One of the participants said:

*“I was taught in school when I was doing my first degree in nursing, but I think the teaching was not well done; so most of us couldn’t understand it. I have been taught; but putting it into practice is a problem”.*

Other participants also indicated that the teaching of the nursing process at school was geared towards the preparation of the student for the licensure examination. Instead of the students learning it well at school; so that they would be able to implement it in the ward after completion of school, they merely memorized it in order to pass their examination without really understanding the main concept. This is what one of the participants had to say:

*“I think that the nursing process is not well understood; because sometimes some of the tutors who teach you about this thing I do not think they know much about it. You know that some of the students treat the nursing process as something that is very difficult because they do not understand it. But if a tutor understands it very well and teaches to the extent that everybody is okay with it, then it will not be difficult for one to implement it.”*

According to the participants, because the nursing process was not made as practical as possible by the tutors who taught it, the students also tended to treat it as theoretical knowledge; and they consequently find it difficult to put the theory into practice. Others also memorized the nursing process and applied the memorized knowledge during the examination – with the aim of passing the examination without – necessarily understanding it. One of the participants had this to say:

*“I think at that time we were just learning it for examination purposes that is how I will put it. You know when you are a student, there is a popular saying like chew pour pass and forget.”*

It is very important for the nursing process to be treated, as one of the most important skills a student nurse needs to acquire – before he or she can complete a nursing training school. The teaching of the nursing process must also be approached with a more-practical attitude. This would equip the student with the practical skills needed to put the acquired knowledge of the nursing process into practice.

### Nurses do not understand the nursing process

The participants indicated that they do not use the nursing process in the ward; because the actual understanding thereof is not there. Others were of the view that the nursing process is a foreign concept; and hence, most nurses do not want to use it. This attitude also influences those who qualified from school recently; because according to them, if you are taught the nursing process in school and you qualify as a nurse; and those that you meet on the ward are not implementing it, with time the newly qualified nurse will tend to forget the acquired knowledge. This was evident in the narrative of two of the participants:

*“The problem was that although we were taught on how to carry out the nursing process at school, when you come to the ward and approach some of the nurses, they seem not to have any idea about what you are talking of because the thing is not being practised in the ward; and therefore, they have forgotten it”(P 1).*

*“I think it is because I do not think they have heard of [the] nursing-care plan. The concept of it I think is not well understood. When you look at those old nurses; you ask somebody about the nursing-care plan; and the person doesn’t know anything about it. If I am a matron and I do not know about how to use the care plan, I do not think I will stress the need for anyone to use it.”*

The nurses’ understanding of the nursing process influenced whether they would implement, it or not. The lack of support from those nurses who were not taught the nursing process – and hence do not know how to apply it – influences the young nurse’s ability to use the nursing process. Although all the participants were professional nurses; some of their seniors they worked with were enrolled nurses; and consequently, they could not help them understand the implementation of the nursing process; since they themselves did not know anything about it.

### The non-availability of the care plan forms in the ward

The participants attributed the non-utilization of the nursing process by nurses in the ward to the unavailability of a nursing-care plan form. According to the participants, they have various documents, which they use to document the care rendered to the patient; and they refer to these documents as the admission papers; and the care plan is supposed to be part of these documents; but for whatever reason, it is not always available in the ward. This non-availability of the care plan in the ward prevents those who would have tried to put the acquired knowledge into practice, not to do so. One participant, who was very passionate in expressing her views on the issues, had this to say:

*“Ideally, we should have care plans where you have all these things written on and then you follow one step and then do the other thing, according to how you have written it down; but I must say I do not remember the last time I saw a care plan in the ward”(P 8).*

Another participant who was eager to say something on this issue also indicated that:

*“I saw the care plan form in school; but in the ward, it is not part of our documents; so you do a general admission of the patient; but then, you don’t plan specially for the patient on a care plan; as you know [it] from school”(P. 5).*

Instead of the care plan being made available in the ward for the nurses to use, in order to successfully implement the nursing process, they stated they only get to see the nursing-care plan form when the students come to the ward for clinical experience. These students bring the care plan along from school – with the objective of learning with it. This was how one of the participants put it:

*“We do not have the care plan in the ward. When the students are in the ward, that is when we get to see the care plan; because we ask them to take patients and plan a care for them”(P 10).*

The non-availability of the nursing-care plan form in the ward was a main factor that prevented the nurses from effectively utilizing the nursing process.

### Use of the nursing process is time-consuming

One of the factors, according to the participants that prevented them from implementing the nursing process, was that they thought it was time consuming. They felt that using the nursing process required a lot of time to be able to properly collect the needed data, in order to plan the care, based on the identified client’s problems. This was also closely linked to the increasing total number of patients that the few nurses were supposed to take care of at a particular point in time. The nurse-patient ratio, according to the participants, was very low, thereby limiting the total amount of time that was at the disposal of the nurses to take care of the patients. This was expressed by the participants in various ways. According to one of the participants:

*“It is time consuming, because of the low nurse-patient ratio. We are dealing with too many patients at a time; and they all need your attention. You keep running around the whole time to set and outline things; so maybe one patient is breathless, you treat that one; and another patient needs your attention urgently; so you cannot go step-by-step; and you need to move on; so it is time and the low nurse-patient ratio”(P 6).*

Another participant also had this to say:

*“It is also time consuming, like I said you have to prepare a care plan for every patient; and when you come on duty, there are a whole lot of things you have to do – so, preparing a care plan for every patient before you start with the actual care will delay the nurse from doing what is supposed to be done for the patient”(P 7).*

The use of the nursing-care plan to document the nursing care rendered to the patient, and the planning of the care before its implementation, was described as time-consuming by the participants. The increasing number of patients that the nurses are expected to care for prevented them from implementing the acquired knowledge of the nursing process.

### Shortage of nurses

The participants mentioned that the shortage of nurses, which was evident in the low nurse- patient ratio, was one of the main factors that prevented them from implementing the nursing process in the ward. Since the nurses were few, the work load of one nurse was overwhelming – so that it prevented them from effectively implementing the nursing process. This was how one of the participants described it:

*“The patients are more than we can manage; the nurses are few. So, if I come on duty and I am the only one to take care of thirty patients, as I told you earlier; sometimes, with the sixty-bed capacity you may be the only professional nurse with two assistants. In this case, considering the nature of the care plan, how many people can you plan a care for at the end of the day? Probably, you will not be able to do much for the patient” (P 3).*

The participants also indicated that due to the shortage of the necessary professional nurses to care for the total number of patients admitted; frequently, they even find it difficult to meet the basic needs of the patients; and this also results in a work overload for the nurses. They easily become tired; and they are not able to provide quality care to the patients. This, therefore, prevents them from implementing the nursing process. This was passionately explained by one of the participants in this way:

*“I am in the medical ward; and more often than not, we have no less than 20 patients in the ward; and you always meet two nurses around; and at times, only one nurse. I mean to be honest, you cannot do whatever you are supposed to do for the patient; because humans as we are, we end up getting tired and you cannot do almost everything that is expected of you; so it is one of the problems that we are facing”(P 4).*

Other participants also indicated that in situations where student nurses come to the ward for clinical experience, their presence usually creates the impression of the presence of adequate staff. But because they are students; they would have to be supervised in whatever they do; and this rather puts more responsibilities on the nurses. Although, with the students, the care plan can be explained, the implementation still becomes a problem; because the student nurses cannot independently carry out the plan. This is what one of the participants had to say:

*“I also think student nurses do support the staff; but these student nurses do not know it all; you can’t delegate them to do some of the duties. You end up doing almost everything. You have so many patients; and you need to follow the nursing orders; so even if the care plan is prepared, the likelihood that you will even go ahead and use it, is very minimal; the reality is that we are short of staff ”(P 8).*

The availability of the required number of professional nurses in the ward in relation to the total number of patients admitted, is very relevant; as far as the implementation of the nursing process is concerned.

## Discussion

The findings revealed that although the nurses were taught the nursing process at school, from their perspective, the nursing tutors who taught them the nursing process did not understand the nursing process themselves. Hence, they could not teach it well for them also to be able to understand it. This finding supports that of Carpenito-Moyet [3], who found that the nursing tutors teaching the nursing process do not understand nursing diagnoses; and therefore, they do not embrace, value, or teach it. According to Carpenito-Moyet [3], the nursing process and the nursing diagnosis are often a visible element in each course syllabus; but in actuality, classroom teachings and discussions are focused mainly on medical diagnoses.

This author observed that too many students spend hours creating care plans by copying from books; they write down the expected care associated with the medical condition over and over; but they fail to learn the critical thinking skills needed for the analysis of the data from their assigned patient. Nursing diagnoses have no relevance for them; and therefore, they are irrelevant after graduation. It was on the basis of these issues, that Carpenito-Moyet [3] suggested that serious attention should be given to the teaching of the nursing process in nursing courses.

The findings from this study revealed that the nurses were not implementing the nursing process in the ward; because they do not really understand it. They attributed the problem to the fact that they were not taught the nursing process very well at school. According to the participants, they saw the nursing process as an academic exercise; and hence, most of them memorized the nursing diagnoses, without really understanding them – with the aim of passing their examinations at school. This finding is in line with that of Ogboukiri as cited by Adeyemo and Olaogun [7], who noted that some nurses found it difficult to implement the nursing process; because some professionals in the health system do not quite understand what it is all about: the changing role of the nurse requires continued competence; and many nurses are unable to maintain a high level of individual competence in nursing practice, skill and knowledge, recognizing and accepting the responsibility for individual action and judgment.

Similarly, in a study on the evaluation and the implementation of the nursing process among nurses, Agunwah [8] found that poor knowledge of the practical use of the nursing process influences the utilization of the nursing process. Also Huguchi, Dulburgen and Duff [9] in their study on the factors associated with the utilisation of nursing diagnoses found that the lack of knowledge on the nursing diagnosis prevented nurses from effectively using these nursing diagnoses.

It was also found in this study, that the nursing-care plan, which is supposed to be part of the admission papers for each patient, was absent from the ward. The only time that most of the nurses in the study got to see the care plan was when the student nurses came into the ward for clinical experience. These students brought the care plan with them, in order to learn from it. This finding is in line with a study by Halverson, Beetcher, Scherb, Olsen, Frost and Orth [10] who revealed that nursing students write care plans for their patients; but this is often done on only one or two patients; and it is seen as only an academic exercise.

Another factor that prevented the nurses from using the nursing process, was that it was perceived as time-consuming. This is in support of the findings by Habermann and Uys [11], who not only found that implementation of the nursing process was time-consuming; but that the nurses were discouraged from using it; because it was seen to interfere with their care of the patient. The findings are also in line with those of Higuchi et al. [9], who reported that time constraints were amongst the factors that prevented the effective use of the nursing process.

Similarly, Agunwah [8] reported that factors, such as lack of time and lack of co-operation among the nurses influenced the utilization of the nursing process. Fernandez-Sola, Granero- Molina, Aguilera-Manrique, Peredo-de Gonzales, Castro-Sanchez and Perez Galdeano [12] also found that lack of time was one of the main threats and obstacles that prevented nurses from properly implementing the nursing process. The findings from this current study are also in line with those of Bjorvell, Wredling and Thorell-Eksstrand [13], who reported lack of time as a major issue in relation to documenting and updating the nursing-care plan.

The above finding is, however, in contrast with the findings by Axelsson, Bjorvell, Mattiasson and Randers [14], who reported in their study on Swedish registered nurses’ incentive to use nursing diagnoses in clinical practice, that the participants did not only state that they were motivated to take the time needed to formulate nursing diagnoses; but they also regarded recording diagnoses as a time-saving tool in nursing practice.

Another finding on the factors that determine the use of the nursing process in the ward was the shortage of professional nurses. This was found to be a very big challenge; even when the nurses wanted to practise what they had been taught, inadequate staff increased the patient-nurse ratio, leading to a work overload; and an inability to put the acquired knowledge into practice. These findings are similar to those of King, Chard and Elliot [15], who in their study on the utilization of nursing diagnoses in three Australian hospitals, concluded that the one main factor that affected the use of the nursing process was understaffing. Also the work of Laryea [16] on the barriers to the implementation of the nursing process found that understaffing was a contributing factor to the non-utilization of the nursing process.

Similarly, Agunwah [8] concluded that the shortage of nurses influenced the utilization of the nursing process. The findings from a cross-sectional quantitative study by Espana and Monsivais [17] revealed that one main barrier to the implementation of the nursing process was work overload. Fernandez-Sola et al. [12] also reported that the high work overload, resulting from a lack of time, was recognized as one of the main obstacles encountered by the nurses, while implementing the nursing process.

The unique findings from this study are as follows: Student nurses memorize the nursing process, in order for them to pass their licence examination – without necessarily understanding it. Furthermore, young nurses who wish to implement the nursing process in the clinical area are not supported by the experienced nurses; since the latter have limited knowledge on it; and additionally, the care plan forms are not readily available in the clinical area, thereby contributing to their non-usage.

The findings could be peculiar to the settings of the study; since the study was conducted in only one hospital.

## Conclusions

The study has provided additional information to nurses on factors impeding their utilization of the nursing process and the strategies to be used in overcoming these challenges; information to Nurse Educators Nursing Training institutions in Ghana on the importance of teaching the Nursing Process to students well and updating their knowledge of nurses on the nursing process. It has further provided a basis for the Ministry of Health, Ghana Health Service and the Nurses and Midwives Council for Ghana to formulate policies that will enhance the utilization of the nursing process in the care of patients; background data for further research on the use of the Nursing process.

### Recommendations

- The care-plan form must officially be made part of the admission papers; and nurses must be made to understand that the admission of patient is incomplete without a completed care plan for the patient.
- Nursing administration should ensure that the care plans and other resources needed by the nurses are regularly and adequately provided.
- There should be periodic workshops and seminars on the nursing process, in order for nursing tutors to equip them with the needed skills and confidence to value and teach the nursing process. This would lead to a better understanding of nursing students on how they can put the acquired knowledge into practice after their completion of school.
- There should be regular in-service training on the nursing process for nurse clinicians, to continually update their knowledge and skills on the nursing process; so that it could be effectively implemented in the ward.

## Additional file

Additional file 1: Interview guide: The additional file consists of the interview guide that was used to conduct the semi-structured interview. (DOCX 14 kb)

## Abbreviations

IRB: Institutional Review Board
NMC: Nursing and Midwifery council
NTC: Nurses’ Training College
