# Supplementary material for: Factors that influence the clinical utilization of the nursing process at a hospital in Accra, Ghana
Source: BMC Nurs. 2017 Jun 9;16:30. doi: 10.1186/s12912-017-0228-0 (PMC5466728; doi:10.1186/s12912-017-0228-0)
Supplement: Additional file 1: — Interview guide: The additional file consists of the interview guide that was used to conduct the semi-structured interview. (DOCX 14 kb) [file 12912_2017_228_MOESM1_ESM.docx]

**Interview Guide**

**Section A**

Socio-demographic data

1. Age
2. Sex
3. Ward
4. Rank
5. For how long have you been practicing as a nurse?
6. How long have you been working on your current ward?

**Guiding Questions**

**Section B (Views of Nurses on the use of the Nursing Process)**

1. How do you plan your care for a patient (E.g. newly admitted or already admitted)?
2. How do you communicate your care to your colleagues?
3. Tell me how you learnt about the nursing process?
4. Tell me how the nursing process is used to manage a patient on the ward?
5. What challenges do you face in the use of the nursing process?
6. Tell me about what you use to plan the care of patients apart from the nursing process?
7. What else do you want to share with me on the use of the nursing process?
